# Disposable bioreactors: from process development to production

**DOI:** 10.1186/1753-6561-5-S8-P2

**Published:** 2011-11-22

**Authors:** Aurore Polès-Lahille, Celine Richard, Sandrine Fisch, David Pedelaborde, Sandy Gerby, Nora Kadi, Virginie Perrier, Robert Trieau, David Balbuena, Laure Valognes, Didier Peyret

**Affiliations:** 1 Merck Serono Biodevelopment, Martillac, France, 33650; 2 Ecole Nationale Supérieure de Technologie des Biomolécules de Bordeaux, Bordeaux, France, 33000

## 

Single-use bioreactors are commonly used for seeding stainless steel bioreactors or for producing material. The profitability of these equipments has been well demonstrated on more that decade. But few data on its scalability have been published.

In 2010-2011, Merck Serono Biodevelopment performed a study in order to evaluate the performance of several disposable bioreactors. As different technologies and scales were available, this study compared the performance of several types of mixing in single-use bioreactors for process development and pilot scale production. In addition, the evaluation was performed for both seeding applications and for clinical material production.

Thus the feature of this study is the comparison of 3 to 50L disposable bioreactors with internal mixing system or external agitation (Table 1). In order to compare their performance with traditional bioreactors, gas flow rate applied in disposable bioreactors was scaled-down from seeding and production stainless steel bioreactors. Furthermore the ratio working volume on total volume and the power input applied to the 3L disposable bioreactor were similar to those in glass bioreactors.

**Table 1:** Description of the different disposable bioreactors assessed.

Name	Mobius^®^ Cellready3L	Nucleo ^TM^	Cultibag STR ^TM^	Cultibag RM ^TM^	Cultibag Orbital ^TM^ β-test
Supplier	Millipore^™^	Pierre Guerin ATMI	Sartorius		
Agitation type	Marine impeller	Paddle	2 x 3-blade segment impeller	Move back and forth	Orbital shaking
Minimum working volume	1L	8L	10L	5.5L	25L
Maximum working volume	2,5L	20L	50L	20L	50L
Oxygen regulation	Micro or marcro sparger	Overlay + Sparger	Overlay + Sparger	Overlay only	Overlay only
pH regulation with carbon dioxide	Headspace or sparger	Sparger	Sparger	Headspace	Headspace
Temperature regulation	Blanket	Jacket	Jacket	Blanket	Blanket

In order to compare the 5 different types of disposable bioreactors, a fed-batch process producing a highly glycosylated molecule was performed. The cell growth during the amplification phase was similar reflected by the doubling time of the cells. It was similar between traditional bioreactors (21h in 250L stainless steel seeding bioreactor) and disposable bioreactors (from 17h in the Nucleo and the Orbital to 19h in the STR)

**Figure 1:**
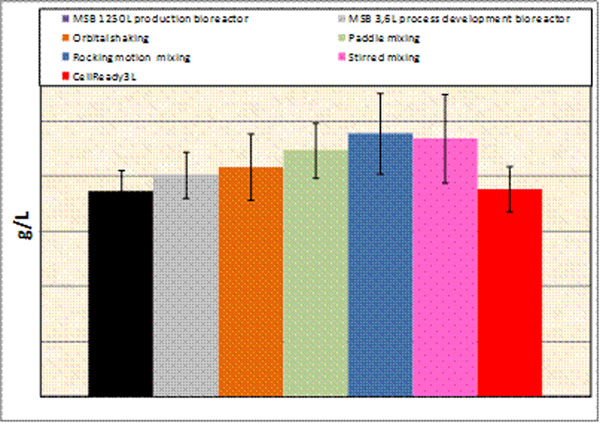
Quantity of highly glycosylated molecule produced in different bioreactors

The quality of the molecule together with the molecule titer and the cell growth were compared in the 5 single use technologies during a production process. The process performance was also compared in 250L and 1.25kL bioreactors and to a 3.6L glass development bioreactor. The overall production process performance was similar in traditional and disposable bioreactors (Figure 1).

This study was completed by a characterization of liquid/liquid and gas/liquid transfers inside each disposable bioreactor in order to estimate their potential in terms of cell culture.

These cells did not require a lot of oxygen. A low k_L_a (0.44h^–1^ in the Nucleo) was sufficient to run this process. The air-flow rates applied in the orbital shaking bioreactor during the process were defined according to Merck Serono Biodevelopment and supplier experience.

These flow rates should have been decreased as k_L_a was higher (3.6h-^1^) than in traditional bioreactors (2.6 h^-1^).

Mixing time was measured in phosphate saline buffer at the maximum working volume and the agitation speed applied during production process. Mixing time was measured by injecting concentrated sodium hydroxide from the top of the bag and from the bottom of the bag.

The Nucleo and the CellReady 3L system have classical probes, while the Orbital, the RM and the STR bioreactors have optical probes. Optical probes were more stable because they took a measure every five seconds. Classical probes were less stable and showed variations of around 0.03 pH unit even a few minutes after injection in the Nucleo. Nevertheless, mixing times on all disposable bioreactors (from 15s in CellReady 3L to 100s in Orbital) were higher than in traditional bioreactors (12s in 3.6L glass bioreactor to 28s in 1250L stainless steel bioreactor). For the STR, the mixing time was close to one minute, which is the recommended maximum for mammalian cell culture. The Orbital and the Nucleo had a mixing time below two minutes. Howerver, after sodium hydroxide injection, pH measured below or close to the final pH value. The disposable bioreactor which has a mixing time closest to traditional bioreactors is the CellReady 3L.

Finally, an evaluation grid was applied to choose the best disposable bioreactor. All these comparisons allowed Merck Serono Biodevelopment to come to a conclusion about disposable bioreactor use and on the scalability (up and down) of these disposable systems. It also enabled us to highlight critical points for disposable bioreactor implementation such as tubing size and length, use of disposable or reusable probes or factory.

